# Reductions in US life expectancy from COVID-19 by Race and Ethnicity: Is 2021 a repetition of 2020?

**DOI:** 10.1101/2021.10.17.21265117

**Published:** 2022-04-05

**Authors:** Theresa Andrasfay, Noreen Goldman

**Affiliations:** a Leonard Davis School of Gerontology, University of Southern California; b Office of Population Research, School of Public and International Affairs, Princeton University

## Abstract

COVID-19 had a huge mortality impact in the US in 2020 and accounted for most of the overall reduction in 2020 life expectancy at birth. There were also extensive racial/ethnic disparities in the mortality impact of COVID-19 in 2020, with the Black and Latino populations experiencing reductions in life expectancy at birth due to COVID-19 approximately two to three times that of the White population. Despite continued vulnerability of these populations, the hope was that widespread distribution of effective vaccines would mitigate the overall impact and reduce racial/ethnic disparities in 2021. In this study, we use cause-deleted life table methods to quantify the impact of COVID-19 mortality on 2021 US period life expectancy by race and ethnicity and compare these impacts to those estimated for 2020. Our estimates, based on provisional COVID-19 deaths for 2021, indicate that racial/ethnic disparities have persisted and that COVID-19 deaths resulted in a decline in life expectancy at birth in 2021 of 1.8 years from 2019, 0.5 years more than estimated for 2020. The corresponding reductions estimated for the Black and Latino populations are 1.3–1.9 times that for Whites, suggesting smaller disparities than those in 2020. However, this narrowing is almost entirely the result of a large increase in COVID-19 mortality among Whites in 2021, in contrast to relatively constant reductions for the Black and Latino populations in the two years. Estimated declines in life expectancy at age 65 due to COVID-19 mortality increased slightly for Whites between 2020 and 2021 but decreased for both the Black and Latino populations, resulting in the same overall reduction (1.1 years) estimated for 2020 and 2021.

## Introduction

**Figure 1.**
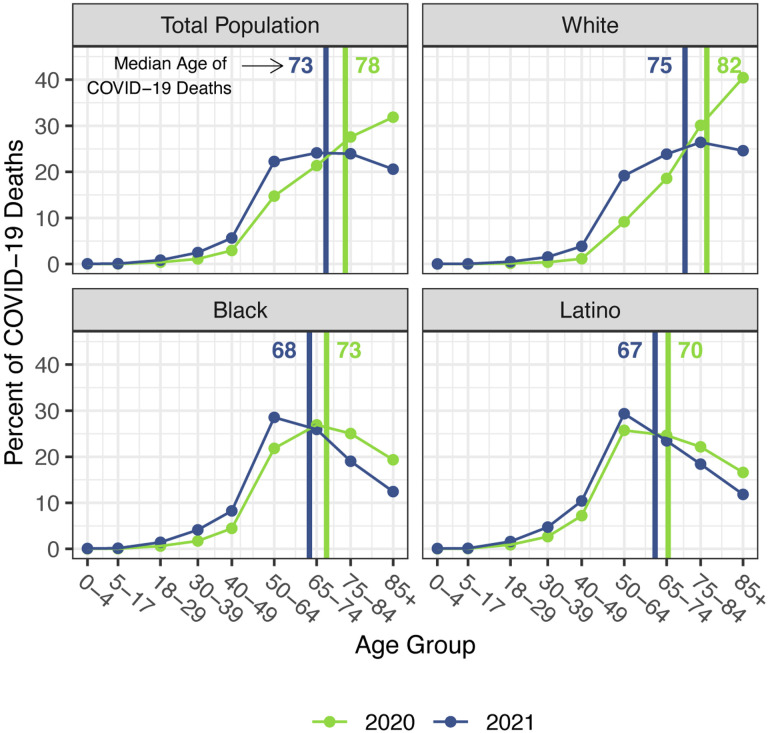
Percentage of COVID-19 deaths in each age group, 2020 and 2021. Vertical lines indicate the median age of COVID-19 deaths in each year. Data are from provisional COVID-19 deaths provided by the National Center for Health Statistics (March 30, 2022, update).

The staggering death toll in the US from COVID-19 has been well-documented: deaths attributed to COVID-19 in 2020 account for most of the 1.8-year reduction in period life expectancy at birth, reversing over 18 years of progress in mortality improvement (1,2). In a previous paper, we predicted that widespread availability of an effective vaccine would lessen the impact of COVID-19 on 2021 life expectancy compared with 2020, although we argued that life expectancy was unlikely to return to pre-pandemic levels (3). Several highly effective vaccines have indeed been developed in record time, but relatively low vaccine coverage in the US, combined with the highly transmissible Delta and Omicron variants of SARS-CoV-2, have led to additional mortality surges, and, by the end of 2021, the total number of COVID-19 deaths had exceeded the 2020 total by 30% (4–6). These sobering numbers combined with the younger age distribution of deaths in 2021 (see Figure 1) – resulting partly from higher vaccination rates among older individuals – indicate that the impact of COVID-19 on US life expectancy in 2021 will be larger than that in the preceding year.

The disproportionate impact of COVID-19 on the survival of vulnerable populations has also received extensive attention: the Latino and Black populations experienced declines in life expectancy over twice as large as that for Whites in 2020 (1). Risk factors for COVID-19 infection and mortality, such as crowded living conditions, frontline jobs with high exposure to infection and low pay, dependence on public transportation, low access to quality healthcare, and high rates of select chronic conditions, still characterize these groups, suggesting continued racial/ethnic disparities in COVID-19 mortality (7–11). A strategically targeted vaccine distribution had the potential to reduce racial/ethnic disparities in COVID-19 mortality in 2021 (12), but many individuals faced barriers to vaccination in the early months, including difficulty scheduling vaccine appointments online, limited supplies, lack of transportation to vaccination sites, and lack of time off work to get vaccinated and recover from side effects (13,14). The resulting inequitable vaccine distribution and uptake may have further exacerbated racial/ethnic disparities in COVID-19 mortality. However, as vaccines became more widely available in the later months of 2021, racial/ethnic differentials in vaccination rates decreased (15,16). By August 2021, differences in vaccination rates by political affiliation, religion, and rural/urban status exceeded racial/ethnic differences (15), suggesting a potential reduction of racial/ethnic disparities in COVID-19 mortality in 2021 relative to those in 2020.

Our goal in the current study is to estimate racial and ethnic disparities in the mortality impact of COVID-19 in 2021 and establish the extent to which these disparities differed from those in the previous year. We do so by calculating reductions in period life expectancy that result from the observed number of COVID-19 deaths for each of these populations. Such period life expectancy reductions are a useful metric during a pandemic because they capture the mortality impact of COVID-19 in a single measure that can be compared across subgroups, nations or years. Specifically, we estimate reductions in life expectancy at birth and at age 65 due to COVID-19 mortality in 2021 for the total population and for the White, Latino, and Black populations in the US. Estimates are based on provisional COVID-19 mortality data for 2021, accessed as of March 30, 2022 (17).

## Methods

Because final all-cause mortality data for the US in 2020 were not available for almost a year after the end of 2020, whereas COVID-19 deaths have been updated regularly throughout the pandemic, timely assessments of the impact of COVID-19 on life expectancy can be estimated based solely on COVID-19 deaths. We use this strategy to estimate the impact of COVID-19 on 2021 period life expectancy (*e*_*x*_): we employ life table techniques developed to estimate the impact on life expectancy of eliminating one or more causes of death (i.e., cause-deleted life tables) (18). In the present study, we assume that mortality conditions in 2021 would be equivalent to those observed pre-pandemic (i.e., had COVID-19 not occurred), and then estimate how the inclusion of COVID-19 deaths alters these mortality conditions. Specifically, we take the 2019 life tables to be cause-deleted life tables in which COVID-19 has been eliminated and recover all-cause life tables for 2021 that incorporate COVID-19 mortality. This strategy has been used in previous studies to estimate the impact of COVID-19 on 2020 life expectancy (3,19–21).

Provisional COVID-19 deaths by age, race, and ethnicity are provided by the National Center for Health Statistics (NCHS) (17). These data include all deaths for which COVID-19 is listed as an underlying, probable, or presumed cause of death, that had been reported to and processed by the NCHS as of March 30, 2022. Mid-year 2021 population estimates by age, race, and ethnicity are obtained from the US Census Bureau (22). Life tables for 2019 for the total US population and for the non-Latino White, non-Latino Black, and Latino populations are obtained from the National Vital Statistics System (NVSS) (23).

We first estimate the expected number of deaths in 2021 in the absence of COVID-19 (_n_D_x_*_,2021_) by multiplying the 2019 age-specific mortality rates (_n_M_x,2019_) by the 2021 mid-year population for the same age range (_n_K_x,2021_). We then estimate the expected number of deaths in 2021 in the presence of COVID-19 (_n_D_x,2021_) by adding the number of COVID-19 deaths in each age group (_n_COV_x,2021_) to the expected number of deaths in each age group from other causes. We assume that individuals who do not die of COVID-19 in 2021 are exposed to the 2019 mortality risks:

Dnx,2021*=Mnx,2019*Knx,2021Dnx,2021=CnOVx,2021+Mnx,2019*(Knx,2021−CnOVx,2021)

We then calculate the age-specific ratio of expected number of deaths in the absence of COVID-19 to expected number of deaths in the presence of COVID-19 (_n_R_x,2021_). Using this ratio and Chiang’s method (18), we adjust the 2019 life table values to reflect the presence of COVID-19 mortality and obtain our estimates of all-cause life tables for 2021. We repeat these calculations for each of the racial/ethnic groups in our study.

Because our cause-deleted life table methodology differs from that used by NVSS to estimate 2020 life expectancy, the magnitude of our 2021 estimates will not be directly comparable to the published 2020 life expectancy estimates from NVSS. To facilitate comparisons between 2020 and 2021, we estimate 2020 life expectancy with the same methods as our 2021 calculations, using the provisional counts of COVID-19 deaths (rather than deaths from all causes) provided by NCHS for all of 2020, and mid-year 2020 population estimates provided by the US Census Bureau. Because these 2020 calculations use updated data on COVID-19 deaths, population size, and pre-pandemic mortality conditions, the estimates differ slightly from those previously published (19).

## Results

**Figure 2.**
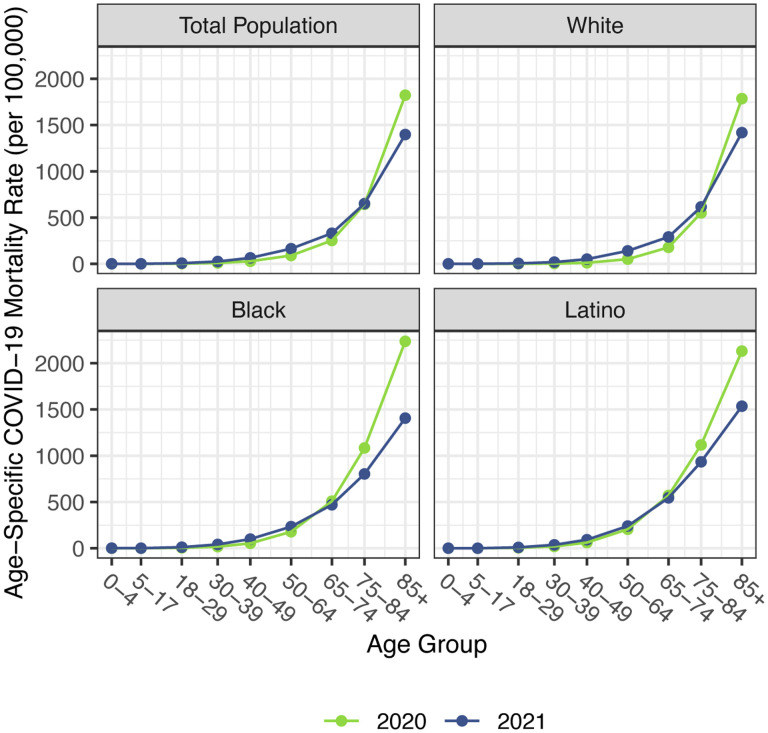
Age-specific COVID-19 mortality rates by race/ethnicity, 2020 and 2021. Estimates are based on provisional COVID-19 deaths provided by the National Center for Health Statistics (March 30, 2022, update).

**Figure 3.**
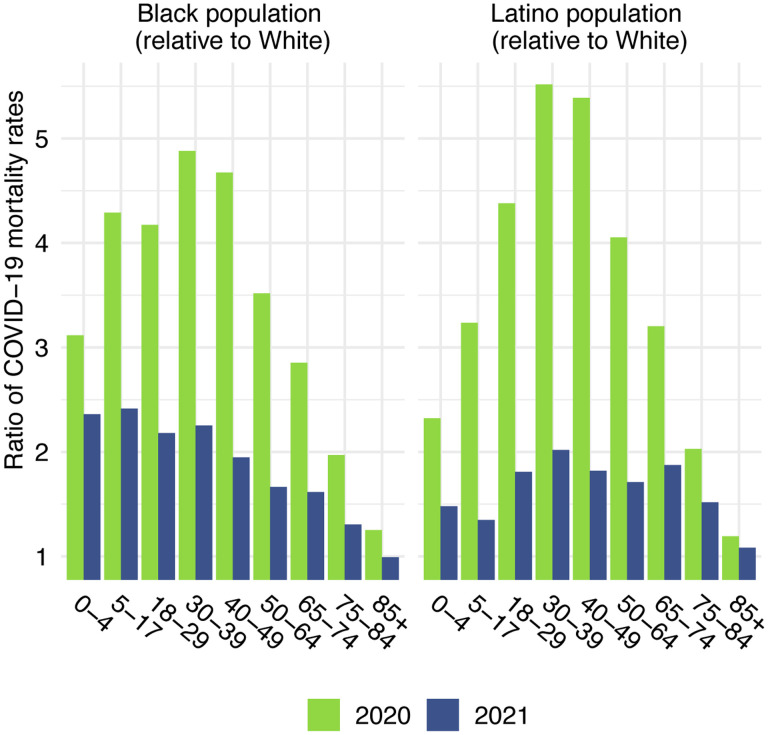
Ratio of age-specific COVID-19 mortality rates relative to the White population, 2020 and 2021. Data are from provisional COVID-19 deaths provided by the National Center for Health Statistics (March 30, 2022, update).

Before presenting estimates of life expectancy during the pandemic, we display descriptive statistics of the age-specific COVID-19 mortality rates, which are defined as the number of COVID-19 deaths in an age group in a given year divided by the total population in that age group at the mid-point of the year. Figure 2, which presents age-specific COVID-19 death rates for each of the past two years, portrays a similar time pattern across racial/ethnic groups: a large decline in rates at the oldest ages between 2020 and 2021, with modest changes – often small increases – below age 65. Figure 3, which shows these rates for the Black and Latino populations divided by the corresponding rates for the White population, highlights the huge decline in relative death rates for the working age population (18–65): COVID-19 death rates at these ages were often three to five times as high in the Black and Latino populations as among Whites in 2020 but roughly twice as high in 2021. These numbers suggest a substantial decline in racial/ethnic disparities in COVID-19 mortality from 2020 to 2021, but the estimates of life expectancy provided below tell a more nuanced story.

Life expectancy estimates for the total US population and by race/ethnicity are displayed in Table 1. All reductions are relative to 2019 life expectancy values, which are displayed in panel A. Our estimates of 2020 life expectancy reductions due to COVID-19 are displayed in panel B; these are estimated using cause-deleted methods, the same procedure used for the 2021 estimates. To highlight the comparisons between 2020 and 2021, Figure 4 displays the magnitudes of the reductions in life expectancy at birth and at age 65 for 2020 and 2021.

Panel C of Table 1 presents the 2021 estimates, based on the provisional number of COVID-19 deaths in 2021. These estimates indicate that COVID-19 deaths in 2021 imply a 1.8-year reduction in life expectancy at birth and a 1.1-year reduction in life expectancy at age 65 for the total US population relative to 2019. In 2021, the reductions in life expectancy at birth are largest for the Latino population (3.1 years), followed by the Black population (2.1 years), and smallest for the White population (1.6 years). The 2021 reductions in life expectancy at birth for the total and White populations substantially exceed the reductions estimated for 2020 (an increase of 0.6 years between 2020 and 2021 for Whites); the increases for the Black and Latino populations are much smaller than for Whites (0.1 and 0.2 years for the Latino and Black populations respectively), but nevertheless do not reveal an expected improvement over the two years. Corresponding estimates for Native Americans presented elsewhere also show a worsening of the mortality impact of COVID-19: a loss in life expectancy at birth that increased from 2.5 years in 2020 to 2.8 years in 2021, i.e., an increase of 0.3 years with losses in both years exceeding those for the Black population (24).

The estimated reductions in life expectancy at birth for the Latino and Black populations are 1.9 and 1.3 times, respectively, the 1.6-year reduction for Whites. Although these estimates of loss in life expectancy relative to Whites are below those in 2020, they reveal another year of pronounced racial/ethnic inequities underlying a huge overall impact of COVID-19 on life expectancy.

## Discussion

With the advent of highly effective vaccines to protect against COVID-19, many in the scientific and public health communities had hoped that the mortality impact of COVID-19 would be substantially lessened in 2021 with period life expectancy converging toward pre-pandemic levels. However, our findings reveal a devasting impact of COVID-19 in 2021, one that is substantially larger overall than that in 2020. This increase occurred even for the Native American population, despite their having vaccination rates that exceeded those for other racial and ethnic groups (24). The disproportionate losses for non-White populations are generally similar to those in 2020, with the Latino population experiencing the largest reduction in life expectancy at birth in 2021 due to COVID-19, approximately one year higher than the Black population.

Our estimates in Table 1 reveal no difference between the decline in life expectancy at age 65 estimated for the total population in 2021 and that estimated for 2020 – the slightly larger impact for Whites in 2021 is counteracted by smaller declines for the Black and Latino populations. This pattern contrasts with the substantial overall decline in life expectancy at birth in 2021 and reflects the shifting distribution of ages at death toward younger ages in 2021 (Figure 1). The younger age distribution of COVID-19 deaths is largely a consequence of the steady increase in the prevalence of vaccination by age, with high levels achieved for the elderly (over 85% of the 65 and over population fully vaccinated by the end of 2021), in contrast to lower coverage among younger adults (approximately 63% of adults aged 25–39 fully vaccinated by the end of 2021) (4). High vaccination rates among nursing home residents, who are particularly vulnerable to adverse COVID-19 outcomes, paired with stricter infection protocols, also helped reduce the mortality impact of COVID-19 on older adults in 2021 (25,26). The net result, as shown in Figure 2, is that mortality from COVID-19 for each racial/ethnic group declined substantially at the oldest ages, but changed relatively little in young and middle age groups. Although differences between 2020 and 2021 in age-specific death rates in young and middle age groups appear small in Figure 2, the modest increases throughout much of this age range, most notable for Whites, make a relatively large contribution to the change in life expectancy at birth between 2020 and 2021.

These results underscore the persistence of large racial/ethnic disparities in the effect of COVID-19 on life expectancy. The disproportionately high losses of life in the Black and Latino populations reflect the social and economic inequities that have been repeatedly acknowledged throughout the pandemic, most notably high rates of poverty and crowded housing, low income jobs that cannot be performed remotely, a high prevalence of chronic health conditions, and inadequate access to quality healthcare (7,8,10). Latinos, who once again appear to have suffered the greatest loss of life from COVID-19, have particularly low levels of health insurance coverage, are more likely to live in multigenerational households than most other groups, and often face language barriers to obtaining comprehensible information on viral transmission and mitigation strategies (8,9,11,27). In addition, Latino workers suffered disproportionate job and income losses during the pandemic because of their overrepresentation in the gig economy and in industries greatly impacted during this period (e.g., construction and leisure and hospitality) and because many Latinos were ineligible for government benefits (28). Although data on race and ethnicity of vaccine recipients are incomplete, existing information suggests that the persistent racial/ethnic disparities are likely partially the result of differences in vaccine uptake early in 2021. For example, after taking differences in age structure into account, Reitsma and colleagues estimate that vaccine uptake rates (for at least one dose) were about 30% higher in Whites than in the Black and Latino populations through the end of March, 2021, with huge variability across states (29).

It is important to emphasize that, although the estimates of life expectancy reductions in Table 1 indicate some narrowing of the differentials from the previous year, this is entirely due to larger life expectancy reductions in the White population rather than to improvements in either the Black or Latino populations. The recent worsening of COVID-19 mortality among Whites could reflect lower adherence to social distancing and masking guidelines relative to other races/ethnicities (30,31). Although all groups reported high adherence to public health guidelines at the beginning of the pandemic, Whites resumed social activities and ceased mask-wearing more quickly than Black and Latino individuals (30,31).

This analysis is subject to several limitations. Our estimates for 2021 life expectancy rely on NCHS provisional COVID-19 deaths, which are subject to reporting and processing delays. Moreover, the cause-deleted life table methodology estimates the impact on period life expectancy of introducing COVID-19 mortality under the assumption that the risk of dying from other causes is not altered by this new illness. As a result, our estimates do not account for excess mortality from causes other than COVID-19 and should not be interpreted as capturing the total impacts of the pandemic on US life expectancy, which will almost certainly be even greater than those shown here.

NVSS estimates of life expectancy loss in 2020, which exceed our estimates based only on COVID-19 deaths, provide insights into the effect of omitting changes in numbers of deaths from other causes (1,2,19). A comparison of cause-specific death rates between 2019 and 2020 indicates a net rise in mortality from non-COVID-19 causes in 2020, often referred to as “excess” deaths: increases in several causes (e.g., drug overdoses and other unintentional injuries, homicides, diabetes, heart disease) had a larger overall impact on life expectancy than decreases in other causes (e.g., cancer, Alzheimer’s disease, chronic lower respiratory diseases) (1,2,32,33). Given that the risk of COVID-19 fatality is increased in the presence of numerous co-morbidities (e.g., cancer, Alzheimer’s disease), mortality rates from some of these chronic diseases may have decreased because severely ill individuals, particularly those with compromised immune systems, succumbed to COVID-19 rather than their underlying condition. In contrast, increased mortality rates from non-COVID-19 causes may have resulted from increased severity of co-morbidities due to COVID-19 infection, delays in primary and preventive care or reduced disease management, and inadequate healthcare due to shortages of equipment, staff, and space (34–37).

It is not yet known whether the impact of the pandemic on non-COVID-19 mortality was lower in 2021 than 2020. If such a change took place, our multiple-decrement estimates will be closer to the overall change in life expectancy in 2021 than in 2020. Although many healthcare facilities resumed close to pre-pandemic levels of operation and some shortages of personnel and supplies were resolved during at least part of 2021, which would have led to a potential reduction in excess deaths from other causes, this could have been counteracted by elevated mortality among those who recovered from COVID-19 (38). Additionally, misidentification or miscoding of cause of death, which could have contributed to either increases or decreases in non-COVID-19 causes of death, may have been lower in 2021 because of increased availability of diagnostic tests.

Despite limitations of the multiple decrement approach, an advantage over estimated losses based on all-cause life expectancy is that we can separately estimate the impact of COVID-19 as a cause of death from the impact of changes in other causes, not all of which are attributable to the new coronavirus. In particular, although the pandemic likely resulted in much of the upsurge in drug overdose deaths in 2020 and 2021, drug overdose deaths had been increasing during the years prior to the pandemic (33). Greater availability and use of synthetic opioids in recent years, especially illicitly manufactured fentanyl and fentanyl analogs which are often mixed or used together with other substances and are especially lethal, have led to a greatly enhanced risk of drug overdose (39,40). In 2020, these external causes of death had a different pattern of racial/ethnic disparities than did COVID-19 deaths. Increases in drug overdose deaths in 2020 were highest among the Black population (33,41), and it is estimated that nearly 20% of the overall 2.9-year reduction in life expectancy at birth for the Black population in 2020 was due to unintentional injuries (including drug overdoses) and homicides (1).

As period measures, our estimates of life expectancy reductions for 2021 quantify loss of life resulting from COVID-19 deaths along with disparities across racial and ethnic groups, but it is important to recognize that they do not represent expectations of remaining life for any living cohort, which depend on future mortality conditions. It is uncertain whether life expectancy in 2022 will show a substantial improvement from the past two years, but there are several reasons for optimism. The administration of additional booster doses should help protect those most at risk of dying from complications of COVID-19 (42,43). Other efforts that should reduce the mortality impact of COVID-19 are the ongoing development of different types of vaccines, including those targeting new and multiple variants, and the increasing availability of effective oral self-administered antiviral treatments, which can reduce the risk that a COVID-19 infection develops into severe disease (44). Many high-income countries that had substantial losses in life expectancy at birth in 2020 experienced less severe reductions in 2021, and some, including France, Belgium, Switzerland, and Sweden, appear to have returned to pre-pandemic life expectancy in 2021 (45).

However, there are also many reasons why 2022 may see continued elevated mortality levels and persistent inequities in the US. At the time of writing in March 2022, all states have either ended or announced plans to end indoor mask mandates as part of a strategy of “living with COVID” or treating it as endemic (46). Such a strategy may entail continued high COVID-19 mortality for several reasons. There is still substantial vaccine refusal in the US, with approximately 15% of adults not having received any dose of a COVID-19 vaccine by the end of 2021 (4), and vaccine refusal is unlikely to be substantially diminished in the near future. The recent appearance of the highly transmissible Omicron variant resulted in another large surge of deaths, despite having lower fatality than previous variants (4,6). There is also a constant threat of additional variants that, as with Omicron, will be at least partly resistant to existing vaccines and perhaps existing treatments.

Apart from direct COVID-19 deaths, there is also evidence that survivors of COVID-19 have increased mortality risks for at least six months following initial recovery (47), and the mortality impact of long COVID is not yet known. In addition, increased risks of dying from a broad range of conditions may have been triggered by detrimental changes in health-related behaviors induced by the many social and economic stressors during the pandemic; these behaviors include higher rates of smoking, drinking and drug use; worse nutrition; and reduced exercise (48–50). And, unfortunately, other long-term impacts of the pandemic on mortality resulting from the many social, economic, and healthcare disruptions during the past two years will likely continue to disproportionately affect vulnerable populations.

## Figures and Tables

**Figure 4.**
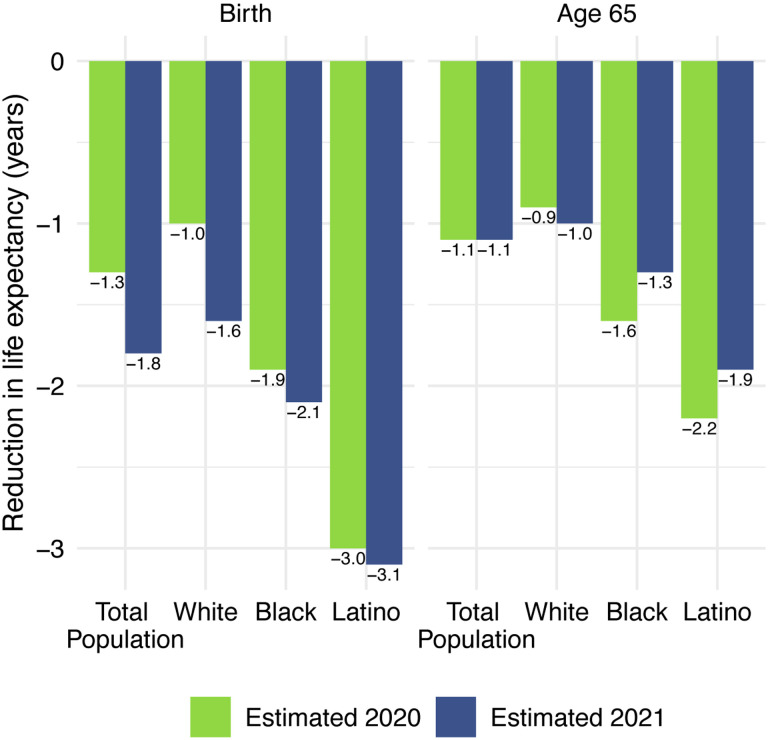
Reduction in life expectancy at birth and at age 65 (years) due to COVID-19 mortality by race/ethnicity, 2020 and 2021. Changes are all relative to 2019 life expectancy. Data are from provisional COVID-19 deaths provided by the National Center for Health Statistics (March 30, 2022, update).

**Table 1:** Life expectancy estimates and reductions from 2019 for the total US population and by race/ethnicity

	Total Population		non-Latino White		non-Latino Black		Latino	
	Birth	Age 65	Birth	Age 65	Birth	Age 65	Birth	Age 65
**A) Pre-pandemic**								
2019 e_x_	78.8	19.6	78.8	19.5	74.8	18.2	81.9	21.6

**B) 2020 estimates**								
Number of COVID-19 deaths	385,477		232,830		61,539		69,474	
Estimated 2020 e_x_	77.5	18.5	77.8	18.6	72.9	16.6	78.9	19.4
Reduction from 2019 e_x_ due to COVID-19	−1.3	−1.1	−1.0	−0.9	−1.9	−1.6	−3.0	−2.2

**C) 2021 estimates**								
Number of COVID-19 deaths	461,539		303,952		61,681		73,201	
Estimated 2021 e_x_	77.0	18.5	77.2	18.5	72.7	16.9	78.8	19.7
Reduction from 2019 e_x_ due to COVID-19	−1.8	−1.1	−1.6	−1.0	−2.1	−1.3	−3.1	−1.9


Notes: Apart from life expectancy (e_x_) values from 2019 that are provided by the National Vital Statistics System, all life expectancy estimates are authors’ calculations. Estimates for 2020 and 2021 are based on provisional COVID-19 death counts provided by the National Center for Health Statistics (March 30, 2022, update).
